# Emergency physician-performed ultrasound-guided nerve blocks in proximal femoral fractures provide safe and effective pain relief: a prospective observational study in The Netherlands

**DOI:** 10.1186/s12245-018-0173-z

**Published:** 2018-03-02

**Authors:** Rein Ketelaars, Joram T. Stollman, Evelien van Eeten, Ties Eikendal, Jörgen Bruhn, Geert-Jan van Geffen

**Affiliations:** 1 Department of Anesthesiology, Pain and Palliative medicine, Radboud university medical center, Geert Grooteplein-Zuid 10, 6525 GA Nijmegen, The Netherlands; 2 Emergency Department, Radboud university medical center, Geert Grooteplein-Zuid 10, 6525 GA Nijmegen, The Netherlands; 3 Emergency Department, Slingeland Hospital, Kruisbergseweg 25, 7009 BL Doetinchem, The Netherlands

## Abstract

**Background:**

The treatment of acute pain in the emergency department is not always optimal. Peripheral nerve blocks using “blind” or nerve stimulator techniques have substantial disadvantages. Ultrasound-guided regional anesthesia may provide quick, safe, and effective pain relief in patients with proximal femoral fractures with severe pain. However, no evidence exists on emergency physician-performed ultrasound-guided regional anesthesia in these patients in Dutch emergency departments. We hypothesized that emergency physicians can be effectively trained to safely perform and implement ultrasound-guided femoral nerve blocks, resulting in effective pain relief in patients with proximal femoral fractures.

**Methods:**

In this prospective observational study, emergency physicians were trained by expert anesthesiologists to perform ultrasound-guided femoral nerve blocks during a single-day course. Femoral nerve blocks were performed on patients with proximal femoral fractures. A system of direct supervision by skilled anesthesiologists and residents was put in place.

**Results:**

A total of 64 femoral nerve blocks were performed. After 30 min, blocks were effective in 69% of patients, and after 60 min, in 83.3%. The mean reduction in pain scores after 30 and 60 min was 3.84 and 4.77, respectively (both *p* <  0.001).

Patients reported a mean satisfaction of 8.42 (1 to 10 scale). No adverse events occurred.

**Conclusions:**

Ultrasound-guided femoral nerve block is an effective, safe, and easy to learn (single-day course) procedure for emergency physicians to implement and perform in the emergency department. Patient satisfaction was high.

## Background

The treatment of acute pain in emergency department (ED) patients is not always optimal [1, 2]. Fortunately, in patients with proximal femoral fractures, peripheral nerve blocks are used increasingly to obtain adequate pain relief [3–6]. In addition to providing pain relief, it may decrease the administration of systemic analgesics such as opioids and decrease their side-effects [7, 8]. Also, undertreated pain and inadequate analgesia have the potential to cause delirium in patients with proximal femoral fractures [9]. Fascia iliaca compartment blocks (FICB) have been performed in hip surgery patients and have shown the potential to reduce the incidence of perioperative delirium in these patients [10].

Nerve blocks in femoral neck fracture patients can be achieved using different techniques. The FICB is a “blind” technique in which surface anatomy landmarks are used to determine the needle insertion point, and tactile feedback guides the correct needle position. Techniques based on surface landmarks have a higher incidence of paresthesia during performance of the block [11]. Furthermore, they produce blocks with a slower onset, lower quality, and shorter duration compared to an ultrasound-guided technique [4, 11, 12]. A nerve stimulator-guided femoral nerve block makes use of electrical nerve stimulation to locate the femoral nerve. If a minimal electrical current still elicits quadriceps muscle contractions, the optimal needle tip position is obtained. Especially in proximal femoral fracture patients, these contractions may be painful and are therefore undesirable [4].

Ultrasound-guided nerve blocks may overcome the aforementioned drawbacks. Ultrasonography allows identification of relevant anatomical structures and continuous needle tip visualization, and even the spread of local anesthetic (LA) may be observed. Ultrasonography in regional anesthesia has increased the success rate and reduced the complications of peripheral nerve blocks [13].

Traditionally, only anesthesiologists performed ultrasound-guided regional anesthesia (UGRA). In recent years, though, emergency physicians (EPs) have been adopting this technique [14–16]. However, in The Netherlands, a lack of evidence exists on EP-performed UGRA in proximal femoral fracture patients.

An ultrasound-guided femoral nerve block and FICB appears to provide quick, safe, and effective acute pain relief and could therefore be a valuable tool adding to current pain management regimes in Dutch EDs [7, 17]. However, EPs should gain relevant knowledge on basic ultrasonography, local anesthetics, nerve block indications, relevant anatomy, block techniques, and complications. Relevant skills to acquire are ultrasound scanning techniques, recognizing sonoanatomy, and ultrasound-guided needle handling. We hypothesize that EPs can be effectively trained to safely perform ultrasound-guided femoral nerve blocks, resulting in effective pain relief in patients with proximal femoral fractures.

## Methods

### Design

A prospective observational study in ED patients with proximal femoral fractures was conducted from June 2014 until June 2017. The aim of the study was to evaluate the effectiveness, safety, and satisfaction of EP-performed ultrasound-guided nerve blocks in the emergency department.

### Recruitment and setting

Adult patients admitted to the Radboud university medical center ED with a proximal femoral fracture, including trochanteric and femoral neck fractures, in whom an ultrasound-guided femoral nerve block or FICB was planned in the ED were included. Exclusion criteria were any sign of infection at the injection site, hemorrhagic diathesis (e.g., hemophilia and use of anticoagulant drugs with international normalized ratio (INR) > 2.0), an allergy to ropivacaine, and multiple traumata.

A 1-day course and an e-learning module were developed by a collaboration of anesthesiologists with extensive experience in UGRA and EPs experienced in general ultrasound. Every EP and EP in training participated in this hands-on course focusing on UGRA of the femoral nerve and FICB. The online pre-course e-learning module and course lectures dealt with basic theory of ultrasound, pharmacology of local anesthetics, indications, relevant anatomy, block techniques, complications and their treatment, and follow-up. Live anatomy and practical block techniques were taught on human cadavers at the Radboud Anatomy Department. Scanning techniques and sonoanatomy were taught and practiced on the participants themselves. Ultrasound-guided needle handling was practiced on blocks of tofu with tiny artifacts inserted.

The UGRA-trained EPs were supervised by skilled (resident) anesthesiologists for five, or more if desired, ultrasound-guided femoral nerve blocks or FICBs until the EPs had gained the knowledge, expertise (as judged by the anesthesiologist), and confidence to perform the procedure independently. A dedicated pool of anesthesiologists and residents with extensive experience in UGRA provided immediate and direct supervision on weekdays during regular business hours. Outside of these hours, supervision was provided depending on the availability of a skilled anesthesiologist or resident.

Before the ultrasound-guided femoral nerve block or FICB was performed, oral informed consent was obtained as part of the standard operating procedure (SOP). No sedative premedication was administered prior to the procedure.

The ED SOP was jointly written by anesthesiologists and EPs, in accordance with the existing SOP for ultrasound-guided femoral nerve blocks performed by anesthesiologists in the operating room. It prescribes a sterile procedure and a conservative maximal LA dosage to minimize risks of local anesthetic systemic toxicity (LAST). Vital parameters (continuous electrocardiogram, pulse rate, oxygen saturation, respiratory rate, and blood pressure) are monitored and continued for 30 min after the procedure. The skin is prepped and draped, and face masks, caps, sterile gloves, and a sterile probe cover are donned. Sterile ultrasound transmission gel and a Stimuplex® Ultra 22G 0.64 × 50 mm, 30°, short bevel (B. Braun, Melsungen, Germany) non-traumatic needle are used. The LA consists of ropivacaine 0.375%, in (four) labeled 10-ml syringes, to allow for a high-volume block without exceeding the maximum dosage. Alternatively, ropivacaine 0.75%, which is also used by anesthesiologists for providing surgical pain relief, can be used. The maximum allowed dose of LA is 2 mg · kg^− 1^ body weight of ropivacaine, injected in 1–2 ml increments under direct ultrasound guidance to confirm the optimal spread of LA around the femoral nerve or below the iliac fascia (FICB). Negative aspiration is confirmed at least every 5 ml to prevent intravascular injection.

In addition to the EPs and EPs in training, all ED nurses were trained to be familiar with the SOP and to be able to assist in the procedures.

The procedure was recorded in the electronic medical record (EMR) according to the SOP. Relevant and additional data were recorded on a dedicated case report form, including gender, age, fracture type and location, and prehospitally administered analgesics (type, dosage, and route of administration). The indication and type of the nerve block, LA dosage, scores on physician and patient satisfaction, pain scores, any rescue medication, adverse events, and any reason to abandon the procedure were also recorded. Vital signs were recorded only in the EMR.

Pain scores were taken in rest on a numeric rating scale (NRS; 0 to 10) on arrival at the ED (t0) and after the procedure at 30, 60, and 120 min (t30, t60, and t120, respectively), but only if still in the ED. We considered a nerve block to be successful whenever there was a pain reduction of at least two points after 30 min compared to baseline. We considered a pain reduction of at least 33% to be clinically important, as inspired by the work of Farrar et al. [18]. An absolute pain score of 4 or less, though, was considered acceptable pain.

Just before discharge from the ED, patients were asked about the level of (dis)comfort they experienced and if they were motivated to undergo a similar procedure in the future. In addition, EPs self-reported five attributes of the procedure. These attributes were scored on a 1–10 rating scale as shown in Table 1.

**Table 1:** Attributes of the ultrasound-guided regional anesthesia procedure

Attribute	Score—1	Score—10
Patient		
(Dis)comfort experienced during the procedure	Very uncomfortable	Not uncomfortable at all
Would like to undergo a similar procedure in the future	Would like it never again	Would like it again
Emergency physician		
Ease of procedure	Very difficult	Very easy
Success of procedure itself regardless of the effect	Did not succeed at all	Very successful procedure
Visibility of anatomical structures on ultrasound	Hard to recognize	Easy to recognize
Spread of local anesthetic on ultrasound	Bad spread	Good spread
Subjective added value of procedure to patient care	No added value	Absolute added value to patient care

These attributes were to be reported by the patients and self-reported emergency physicians on a 1–10 numeric rating scale

### Statistical analysis

Normally distributed data are reported as mean ± standard deviation (SD) or 95% confidence interval (CI), and data with a skewed distribution, including absolute pain scores, are reported as median with an interquartile range (IQR). A one-tailed paired Wilcoxon test was used to test for differences in pain scores within subjects because these are ordinal values, not normally distributed. To test for differences between subgroups in relative and absolute pain score changes (normally distributed), a *t* test and a one-way ANOVA was used. Pearson correlation was calculated for the influence of age and injected volume of LA. Statistical significance was considered at *P* <  0.05. For statistical analysis, IBM SPSS Statistics for Windows, version 22.0 (IBM Corp., Armonk, NY, USA), and GraphPad Prism version 5.00 for Windows (GraphPad Software, San Diego, CA, USA) were used.

## Results and discussion

In total, 64 patients (23 males, 41 females) received UGRA by EPs in the ED for a proximal femoral fracture. Demographics, types of fractures, and prehospitally administered analgesics are displayed in Table 2.

**Table 2:** Demographics and type of fracture

Factors	Frequency or value	%	Range
Median age (IQR), year	76 (68–84)		28–95
Gender			
Male	23	35.9	
Female	41	64.1	
Type of fracture			
Femoral neck	37	57.8	
Trochanteric	16	25.0	
Femoral shaft	11	17.2	
Laterality			
Left femur	30	46.9	
Right femur	34	53.1	
Prehospital analgesics			
Rate of administration	51	79.7	
Paracetamol/acetaminophen	19	37.3	
NSAIDs	0	0	
Oxycodone	1	2.0	
Fentanyl	26	51.0	
Morphine	6	11.8	
Esketamine	14	27.5	

*IQR* interquartile range, *NSAIDs* nonsteroidal anti-inflammatory drugs

Fourteen EPs and EPs in training performed on average 4.6 (CI 3.1–6.0) ultrasound-guided nerve blocks of which 13 (20.3%) were FICBs and 51 (79.7%) femoral nerve blocks.

Ropivacaine 0.375% was used in 28 (43.8%) patients with a mean volume of 22.8 ml (± 6.6 ml, range 13–40 ml). Ropivacaine 0.75% was used in 36 (56.3%) patients with a mean volume of 18.9 ml (± 2.9 ml, range 10–20 ml). All blocks proceeded uneventfully, although one patient with mild pain on injection and one event of transient periprocedural hypotension, unrelated to LA injection, were reported.

### Patient perspective

**Fig. 1:**
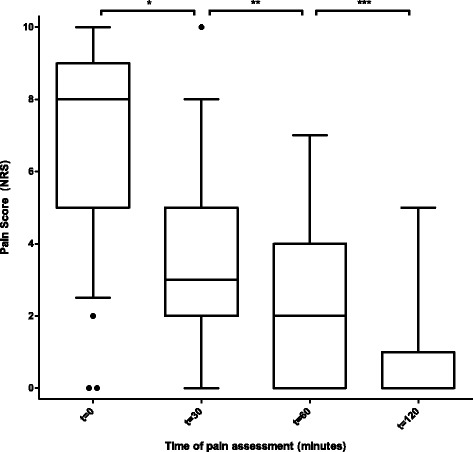
Pain scores at baseline and after emergency department ultrasound-guided regional anesthesia in proximal femoral fractures. Pain scores at baseline and at 30, 60, and 120 min after an emergency physician-performed ultrasound-guided nerve block in emergency department patients with a proximal femoral fracture. 0 = absolutely no pain; 10 = most extreme pain. Boxes show median and interquartile range, whiskers mark the minimum and maximum (1.5 × lower and upper quartile), and dots are outliers. *NRS* numeric rating scale. *p <  0.001; **p <  0.001; ***p = 0.03

The median pain score on an NRS at baseline (t0) before UGRA was 8 (IQR 5–9). At 30 min (t30) after the nerve block, the median score was significantly reduced to 3 (IQR 2–5, *P* <  0.001). The pain was significantly reduced even further to 2 (IQR 0–4) at t60 and to 1 (IQR 0–1) at t120. Relative and absolute pain reductions are shown in Table 3 and Fig. 1.

**Table 3:** Pain scores and reduction from baseline at 30, 60, and 120 min

	*N* (%)	Pain score, median (IQR)	Pain score ≤ 4 *n* (%)	Pain reduction ≥ 33% *n* (%)	Pain reduction	CI	*p* value
Baseline	64 (100)	8 (5–9)	6 (9.4%)	–	–		
t30	58 (90.6)	3 (2–5)	40 (69.0)	41 (70.7)	3.84	3.15–4.54	< 0.001
					50.9%	42.6–59.2	< 0.001
t60	30 (46.9)	2 (0–4)	25 (83.3)	24 (80.0)	4.77	3.73–5.80	< 0.001
					64.4%	52.1–76.8	< 0.001
t120	7 (10.9)	1 (0–1)	6 (85.7)	6 (85.7)	5.85	2.72–8.99	0.002
					79.5%	46.3–100.0	< 0.001

Median pain scores at baseline, 30, 60, and 120 min. Pain scores of 4 or lower, pain reduction within subjects of at least 33%, overall pain reduction relative, and in numeric rating scale (NRS) points including 95% confidence interval (CI) and *p* value

Patient-reported (dis)comfort was scored a median of 8 (IQR 8–9, *n* = 61). When asked if they were motivated to undergo a similar procedure in the future, the score was 9 (IQR 8–10, *n* = 60).

Pain reduction was not found to be significantly related to gender, age, fracture types, prehospital administration (and type) of analgesics, the two block types, the two different concentrations of ropivacaine, or volume of injected LA. Also, we found no significant difference in pain reduction at t30, t60, and t120, whether the EPs performed the blocks with or without supervision. As shown in Table 4.

**Table 4:** Influence of relevant factors on absolute pain reduction 30 min after ultrasound-guided regional anesthesia

Factors	Mean difference	95% CI	*p* value
*t* test			
Gender (male, female)	− 0.03	− 1.48–1.42	0.97
Laterality (left, right)	− 0.55	− 1.95–0.85	0.43
Prehospital analgesics (no, yes)	0.96	− 3.56–5.47	0.57
Block type (femoral nerve, FICB)	1.25	− 0.32–2.81	0.11
Ropivacaine concentration (0.375%, 0.75%)	− 0.13	− 1.56–1.30	0.43
One-way ANOVA			
Fracture type (femoral neck, trochanteric, femoral shaft)			0.10
Correlation	Pearson’s *r*		
Age	− 0.063		0.32
Volume of ropivacaine 0.375%	− 0.230		0.14
Volume of ropivacaine 0.75%	− 0.078		0.33

Mean difference is without dimension because it is the absolute reduction in pain score on a 0–10 numeric rating scale. A positive difference indicates the pain score reduction is larger in a variable’s second value

*FICB* fascia iliaca compartment block

### EP perspective

The 1-day course was very well received, and the EPs were enthusiastic about starting to perform the nerve blocks after the course. The median scores, IQR in parentheses, of the self-reported qualifications of the procedure (*n* = 59) were ease of procedure 8 (7–9), success of procedure itself, regardless of the effect 9 (8–10), visibility of anatomical structures on ultrasound 8 (7–9), spread of local anesthetic 9 (8–10), and subjective added value of the procedure to patient care 9 (8–10).

### Discussion

We showed that, after having received appropriate training, Dutch EPs are able to safely perform UGRA in ED patients presenting with a proximal femoral fracture and severe pain. After 30 and 60 min, mean pain reduction was respectively 3.84 (50.9%) and 4.77 (64.4%). A pain score of 4 or less was reported by 69 and 83.3%, respectively, of the patients. The EPs thought the procedure was easy to perform, and they were able to obtain a good visualization of the relevant anatomy and LA spread. This means that performing a peripheral nerve block is an effective pain relief strategy provided by the very first physician they encounter upon admittance in the hospital.

To date, there are only few similar reports of EPs performing UGRA in the ED. At 30 and 60 min after the nerve block, we found a meaningful pain reduction, respectively, in 70.7 and 80.0% of the patients. We could confirm the results reported by Groot et al. who reported that on a Dutch ED, EP-performed blind FICBs were safe and effective. In 26 of 34 patients (76%), they found a clinically meaningful pain score reduction after 120 min [19]. An explanation for the slightly better and faster pain reduction in the present study might be that we have used an ultrasound-guided technique, compared to their blind FICB. Accurate deposition of the LA in relation to the fascia iliaca, or adjacent to or surrounding the femoral nerve will lead to a more effective and faster effect.

Dochez et al. described the effect of blind FICBs performed by EMS nurses in 100 patients with suspected proximal femoral fractures. After 30 min, they reported a successful block in 96% and median pain scores were reduced from 8 to 3, and on arrival at the ED, 75% had a pain score of 4 or less [20]. Median pain reduction at *t* = 30 and pain scores of 4 or less were comparable (respectively, 5 and 69.9%).

Gozlan et al. described prehospital EP-performed blind FICBs in 52 patients with femoral fractures and reported a success rate of 94% and pain reduction comparable to Dochez and the present study [21]. Morrison et al. compared standard analgesics with EP-performed ultrasound-guided femoral nerve blocks and found a significant difference in favor of the latter. Baseline NRS was 6.4 in both groups and decreased to 5.3 and 3.5, respectively, after 2 h. Unfortunately, they did not report within-subject pain score reduction in the nerve block group [15].

A study by Beaudoin et al. reported similar results to our study [17]. Thirty-six ED patients with proximal femoral fractures were randomized between a femoral nerve block and conventional analgesia. The median pain score in the nerve block group reduced from a mean NRS of 8 at baseline to 4 after 4 h. In the group receiving standard care, the NRS was 8 initially and showed no improvement [17]. Haines et al. described 20 ED ultrasound-guided FICBs performed by six EPs, fellows, and residents. They found a pain reduction from a mean NRS of 7.9 at baseline to 2.05 at 30 min and 1.30 at 120 min [22].

We showed EPs and EPs in training can be taught to effectively and safely perform UGRA in the ED. In addition, they experienced the procedure to be relatively easy and successful, represented by the high EP-reported scores for success, easiness, visibility of the anatomy, and quality of LA spread. These findings implicate that (Dutch) EPs should consider the introduction of ultrasound-guided regional anesthesia in proximal femoral fracture patients in the ED to provide superior pain management as compared to conventional systemic analgesics. Such a program should preferably be introduced in cooperation with their colleagues from the anesthesiology department. Once a successful UGRA program for these patients is implemented, it can be extended to other indications in need of excellent pain management.

With this project, we followed the recommendation by Wu et al. that anesthesiologists with extensive experience in regional anesthesia should introduce these techniques into settings outside the operating room and in the early treatment phases of trauma patients to provide the benefits of regional anesthesia [23]. Although in our institution, skilled anesthesiologists collaborated with EPs to successfully introduce UGRA in the ED, this approach might not be feasible in other comparable hospitals. EPs must connect with instructors with sufficient skills who are willing and able to invest their time and energy. Nevertheless, superior pain relief should be obtained in trauma patients as early as possible, preferably by the first—prehospital—care provider they encounter [24]. If this *journey* cannot start at home, adequate pain relief needs to be taken care of by the first care provider they encounter in the hospital.

This study adds to the literature because we took a unique approach in the introduction and execution of these blocks on our ED through a productive collaboration between the two (ED and anesthesiology) departments.

Furthermore, to date, there have been no published reports of effective and safe UGRA in a Dutch ED.

### Limitations

This study has several limitations. The observational study design is not optimal to answer the main question. A relatively small number of patients was included based on convenience sampling. The aim was to treat proximal femoral fracture patients with ultrasound-guided nerve blocks as the treatment of choice. Although UGRA was recently introduced in our ED, its effectiveness has been proven extensively [8, 13, 25]. To guarantee efficacy and safety of the blocks in the ED setting, anesthesiologists have trained the EPs and supervised the blocks on request. Therefore, we chose not to compare with traditional treatment strategies but to investigate pain reduction within subjects and explore subjective experiences of both health care providers and patients. Another limitation is the increasing amount of missing data at the 30-, 60-, and 120-min intervals from baseline. This might be caused by high ED work-load, resulting in incompletely filled-out case report forms, but is mainly due to expeditious patient transfer to the ward or the operating room. Also, there is a risk of bias because the EPs and ED nurses who performed the blocks filled out the case report forms themselves. These limitations can be partly justified because the efficacy of these blocks has been proven in general. In the present study, they have been performed in most (78%) cases by at least two physicians (or residents) and an ED nurse all verifying the correct technique.

## Conclusions

In conclusion, this study demonstrates that, through close cooperation between EPs and anesthesiologists, after a 1-day training (Dutch), EPs can learn to safely perform ultrasound-guided nerve blocks in proximal femoral fracture patients in the ED, resulting in effective acute pain relief.

## Abbreviations

ANOVA: Analysis of variance
CI: Confidence interval
ED: Emergency department
EMR: Electronic medical record
EP: Emergency physician
FICB: Fascia iliaca compartment block
IQR: Interquartile range
LA: Local anesthetic
LAST: Local anesthetic systemic toxicity
NRS: Numeric rating scale
NSAID: Nonsteroidal anti-inflammatory drug
SD: Standard deviation
SOP: Standard operating procedure
UGRA: Ultrasound-guided regional anesthesia
